# Supplementary material for: Genomic architecture of adaptive radiation and hybridization in Alpine whitefish
Source: Nat Commun. 2022 Aug 2;13:4479. doi: 10.1038/s41467-022-32181-8 (PMC9345977; doi:10.1038/s41467-022-32181-8)
Supplement: Supplementary file 1 — Supplementary Information [file 41467_2022_32181_MOESM1_ESM.pdf]

Supplementary information

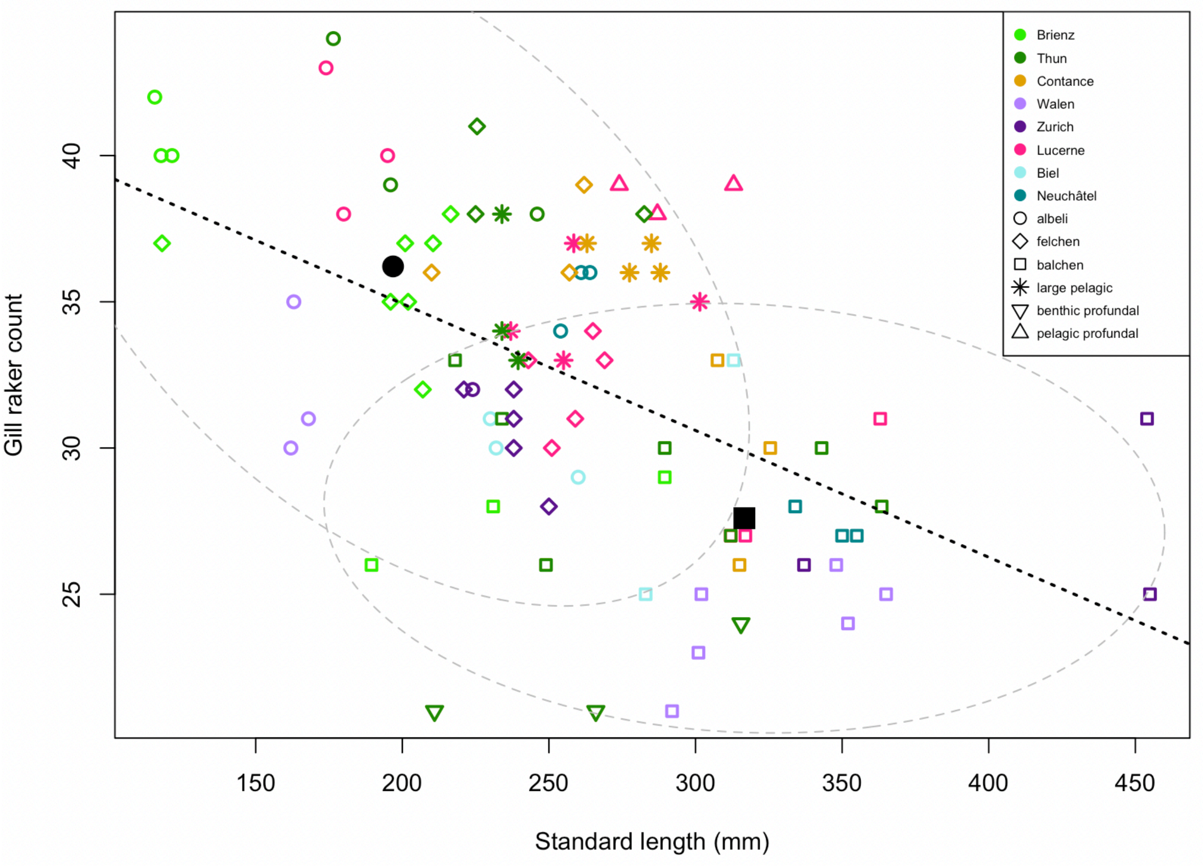

Supplementary Figure 1. Standard length and gill raker count of whitefish were correlated across the dataset (black line indicates linear regression; R^2=0.2767, p=1.2x10^{-7}). ‘Albeli’ species (circles) tend towards high gill raker counts and smaller standard length, and ‘Balchen’ species (squares) tend towards lower gill raker counts and larger standard lengths as indicated by centroids (black solid circle and square denoting the mean value of all ‘Albeli’ and ‘Balchen’ individuals, respectively) and 95% confidence ellipses (grey) for each ecomorph. Mann-Whitney-U tests confirmed that ‘Balchen’ individuals across the dataset have significantly longer standard lengths than ‘Albeli’ (W=33, p=2.457x10^{-7}, Albeli n=19, Balchen n=30) and that ‘Balchen’ individuals have significantly lower gill raker counts than ‘Albeli’ individuals (W=533.5, p =3.351x10^{-7}, Albeli n=19, Balchen n=30). The legend indicates the ecomorph of each individual and lake it is from, with colours and symbols used consistently with Figure 1.

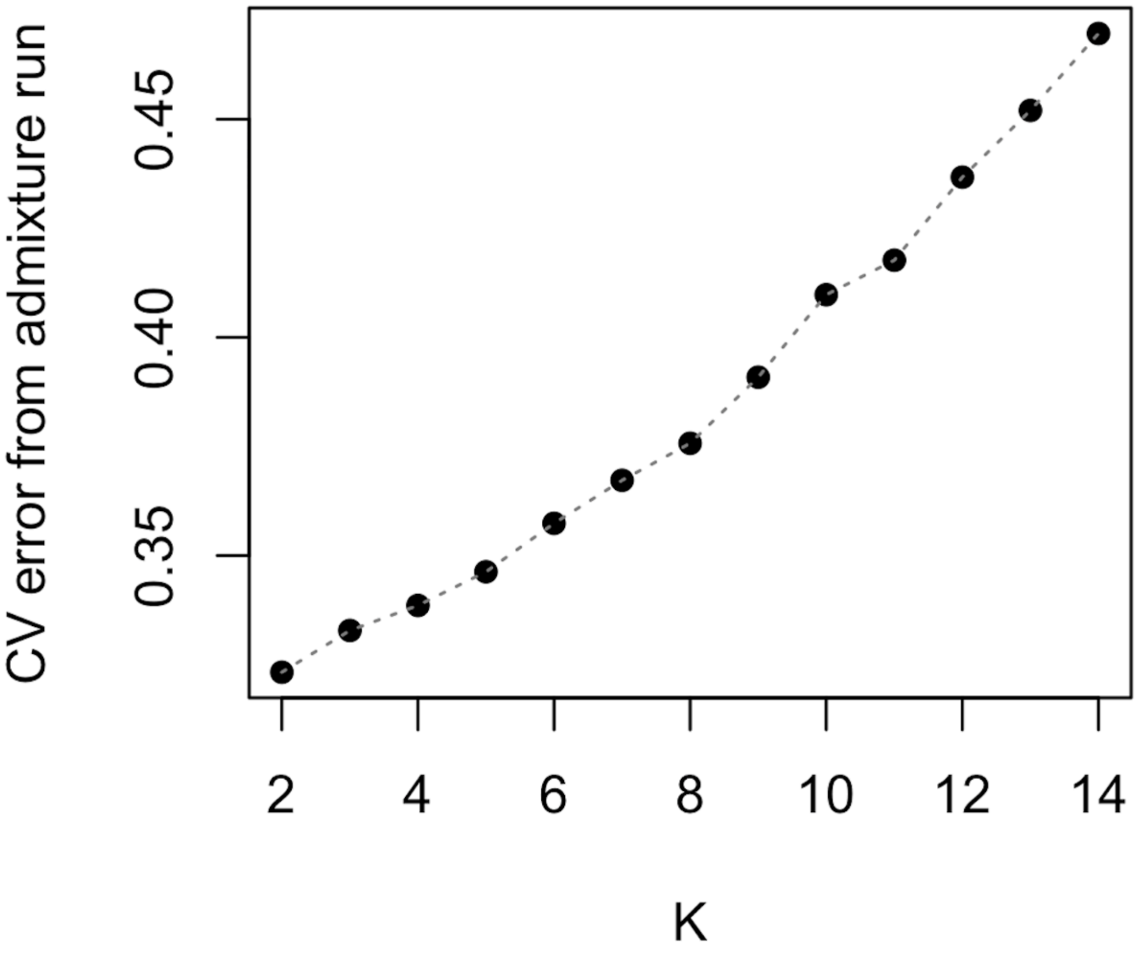

Supplementary Figure 2. CV error increased with the number of populations (K) for each admixture run. Lower values of K resulted in lower CV error, and higher values of K resulted in higher CV error. K=7 was selected for plotting since it helped identify lake-specific differences.

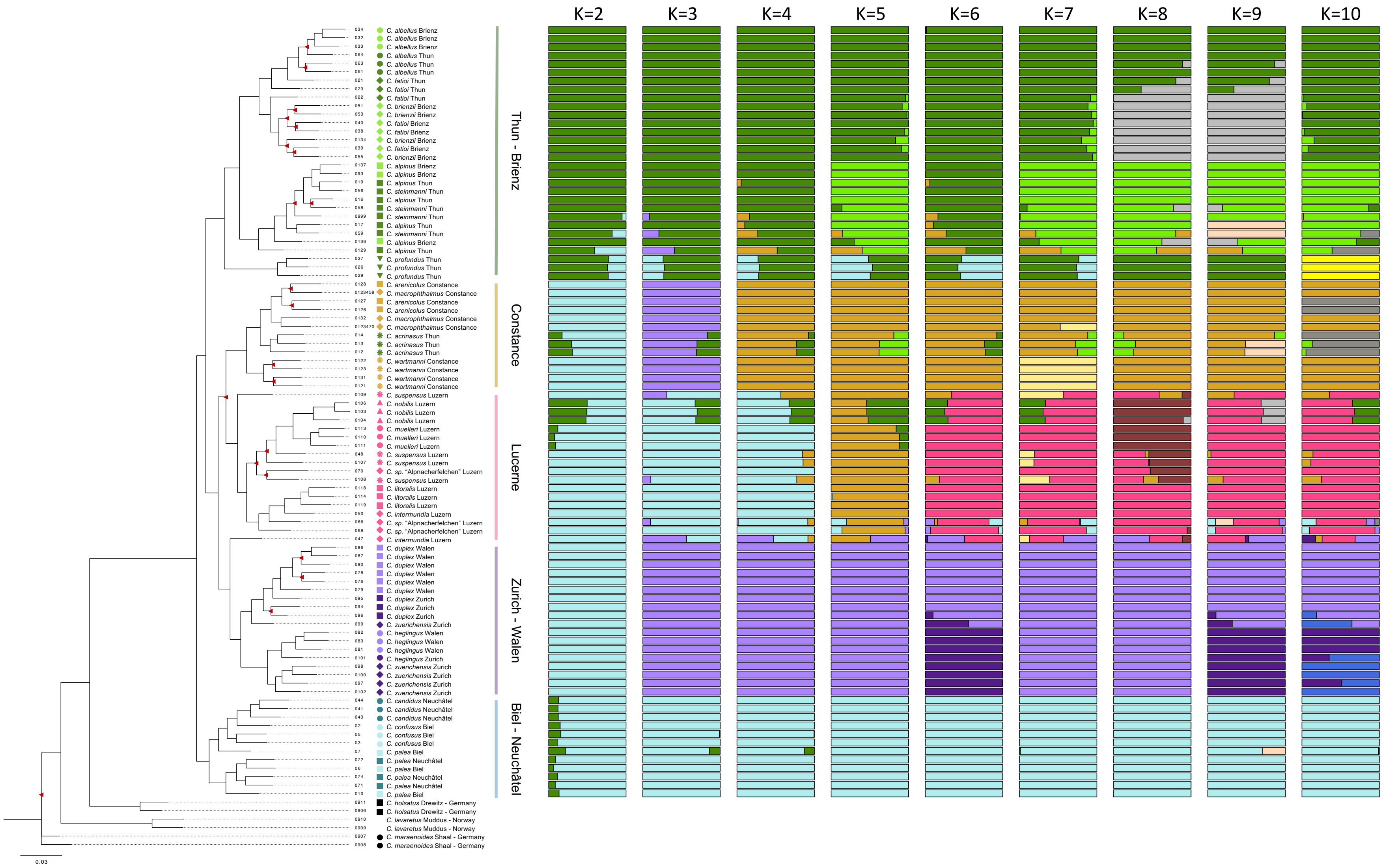

Supplementary Figure 3. Our maximum likelihood RAxML phylogeny (as in Figure 1) with admixture analysis where the number of populations (K) ranges from 2 to 10. Increased values of K resulted in more complex admixture patterns, but between-lake population structure remained relatively consistent.

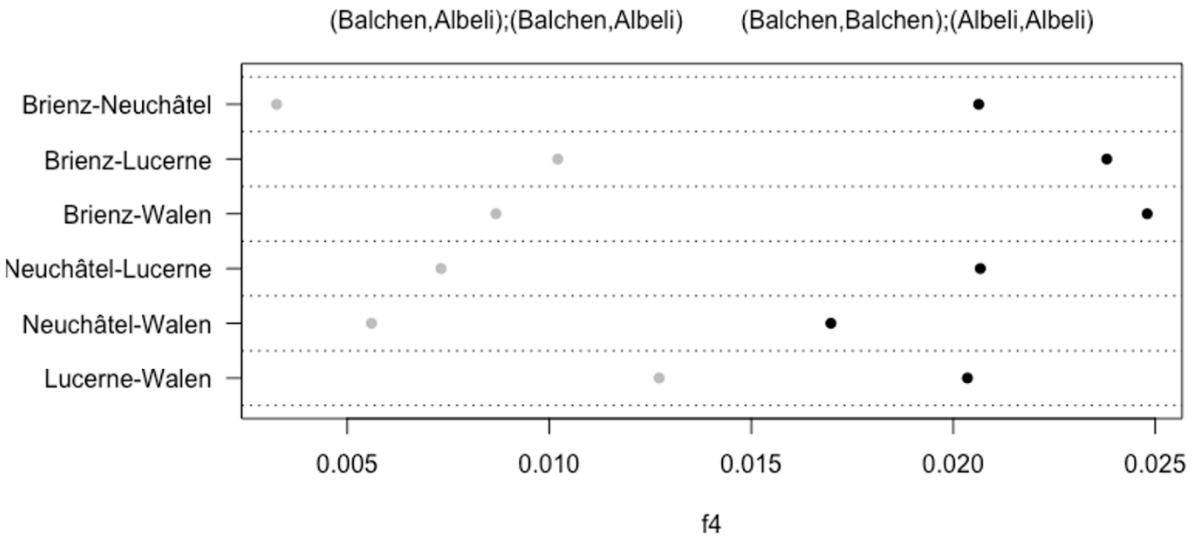

Supplementary Figure 4. F4 statistics calculated for topologies where 'Balchen' and 'Albeli' for each of the lakes Brienz, Neuchâtel, Walen, and Lucerne where either sister to one another i.e. sorted by lake (grey), or where species of the same ecomorph are sympatric to one another regardless of lake (black). f4 statistics of the topology inconsistent (right, black) with the phylogenetic tree of the adaptive radiation (Figure 1) are higher than f4 statistics consistent with its topology (left, grey), supporting a scenario in which 'Balchen' and 'Albeli' ecomorphs evolved independently in parallel in each pre-Alpine lake (see Methods and Supplementary Table S1 for more details).

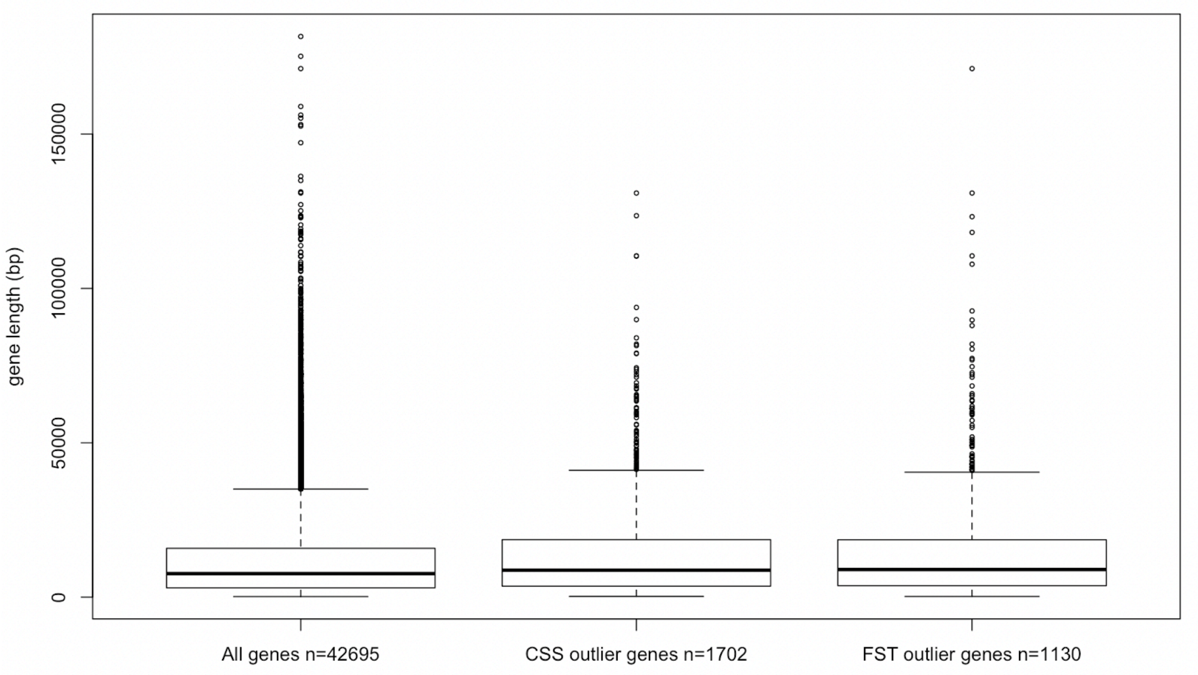

Supplementary Figure 5. Boxplot showing the length distributions of the 1702 genes that overlapped with our CSS outlier windows and 1130 genes that overlapped with F_{ST} outlier windows. The centre of the box represents the median gene length for each gene set, the top and bottom bounds of the box represent the third and first quantiles of the distribution, respectively. The uppermost whisker extends to 1.5x the interquartile range, with maxima lengths beyond this represented by outlier points, and the lowermost whisker represents the minima gene lengths in each set. Genes overlapping with CSS outlier windows were significantly longer than those that did not (Mann-Whitney-U test; one-sided; W = 32178234, p-value = 2.775×10^{-8}; for genes overlapping with CSS outlier windows n = 1,702 and for those that did not n = 40,993). Similarly, genes overlapping with F_{ST} outlier windows were significantly longer than those that did not (Mann-Whitney-U test; one-sided; W = 21624555, p-value = 2.693×10^{-6}; for genes overlapping with F_{ST} outlier windows n = 1,130 and for those that did not n = 41,565).

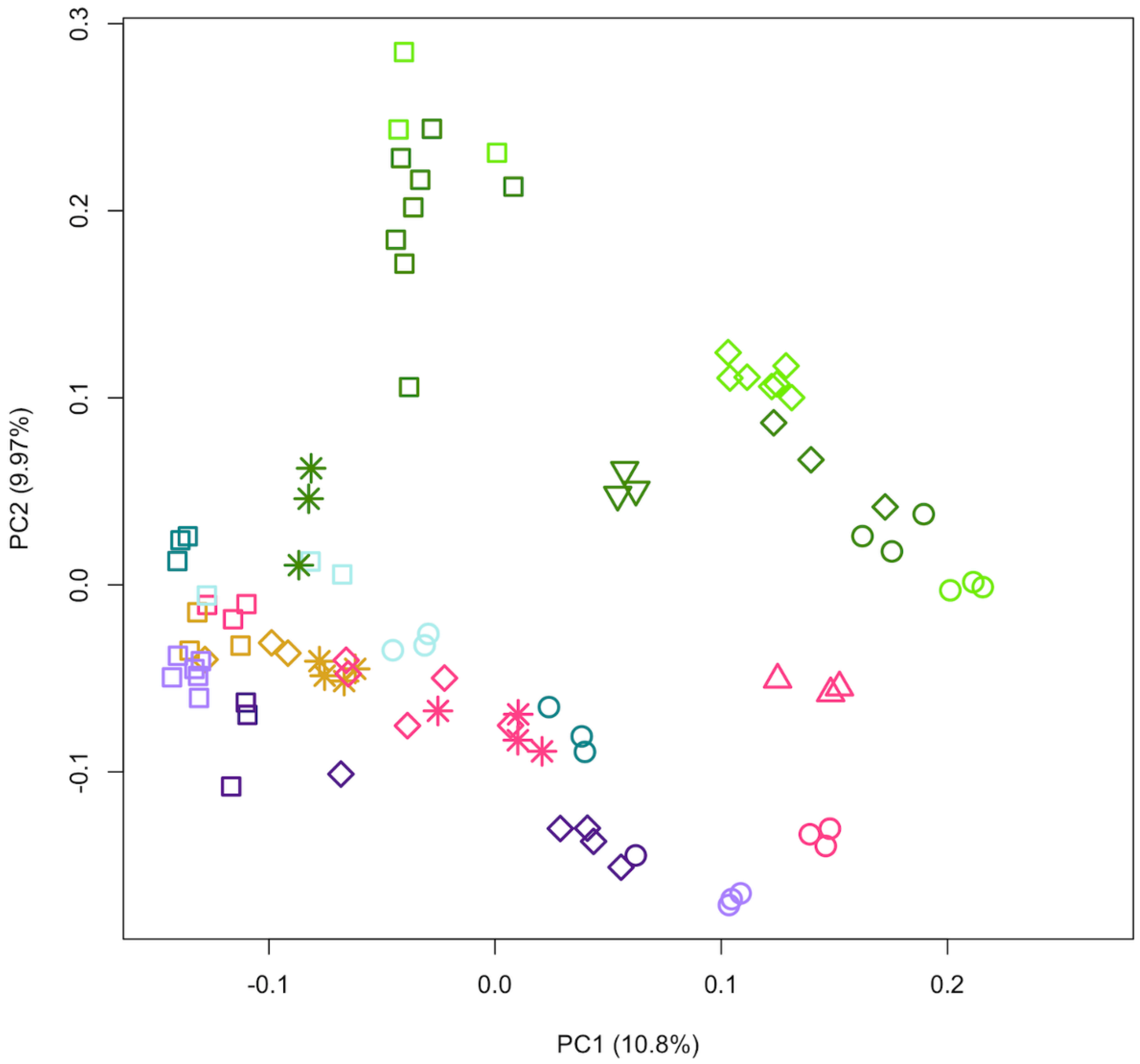

Supplementary Figure 6. In our PCA including only linkage-filtered SNPs within the 1659 CSS outlier windows PC1 separates out species and PC2 separates out the Thun/Brienz lake-system from all four other lake-systems. Symbols are used as in Supplementary Figure 1 and Figure 1.

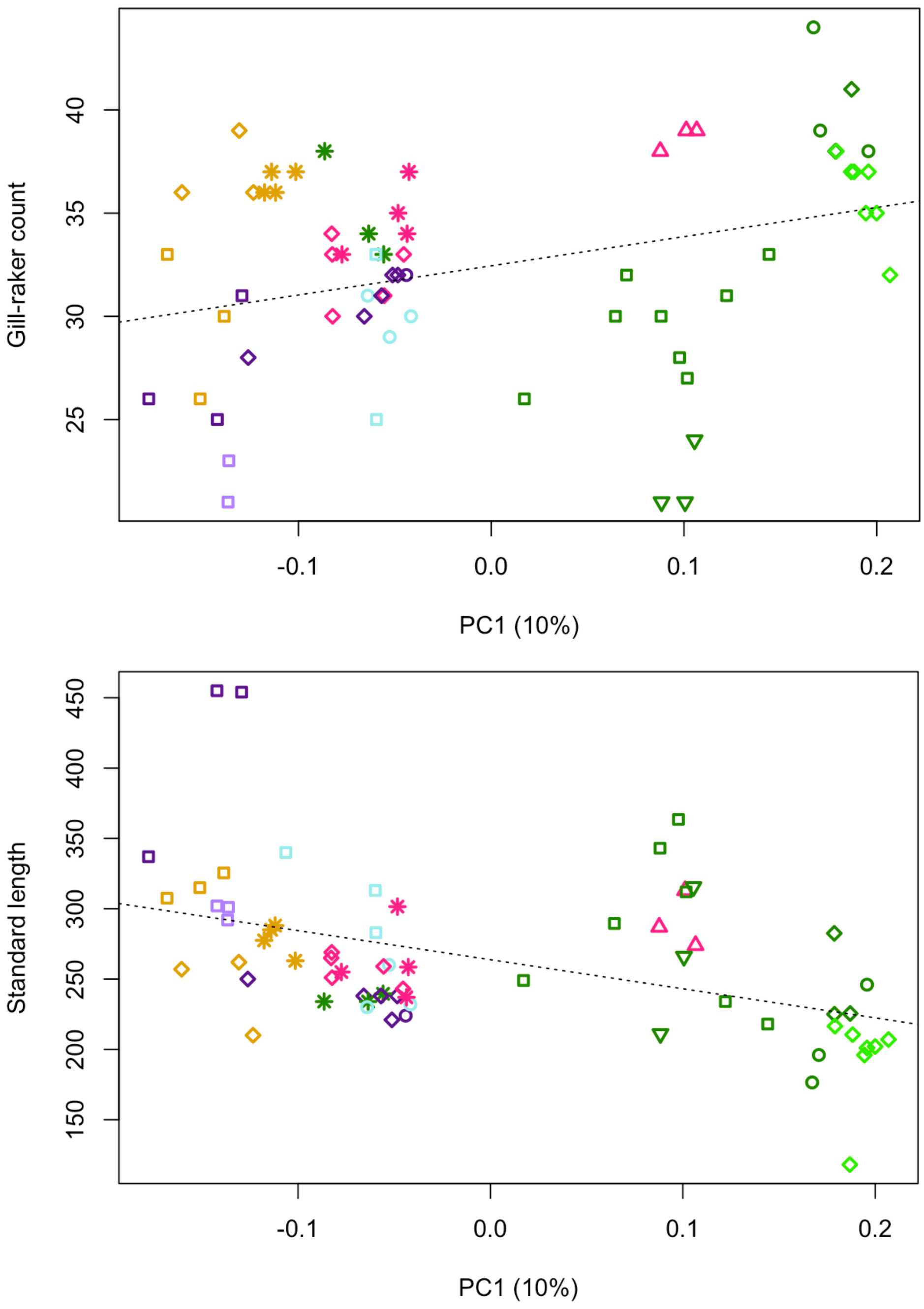

Supplementary Figure 7. When the 24 samples used for the CSS analysis were excluded prior to PCA construction correlations between PC1 and gill-raker count (top), R2=0.114, p=0.005667, and PC1 and standard length (bottom) R2=0.208, p=1.183x10-4 were still significant. Symbols are used as in Supplementary Figure 1 and Figure 1.

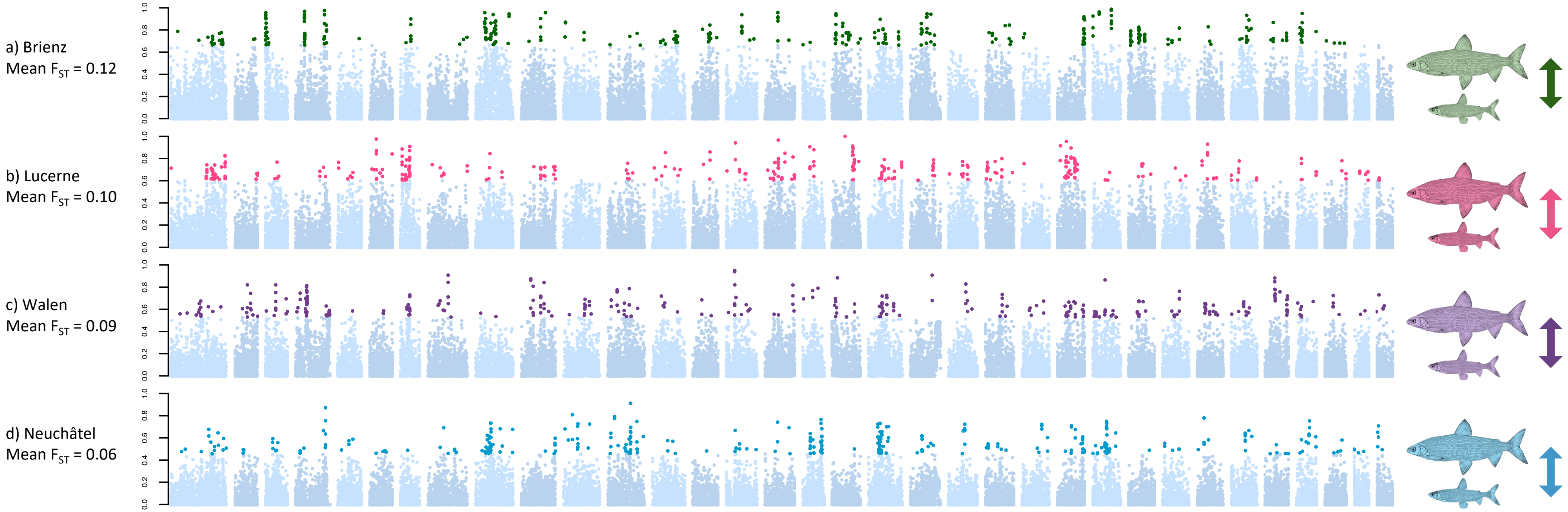

Supplementary Figure 8. Pairwise F_{ST} scans (50 kb windows) for 'Balchen' and 'Albeli' species from Lakes Brienz (a), Lucerne (b), Walen (c), and Neuchâtel (d). Mean genome-wide F_{ST} is shown for each pairwise comparison.

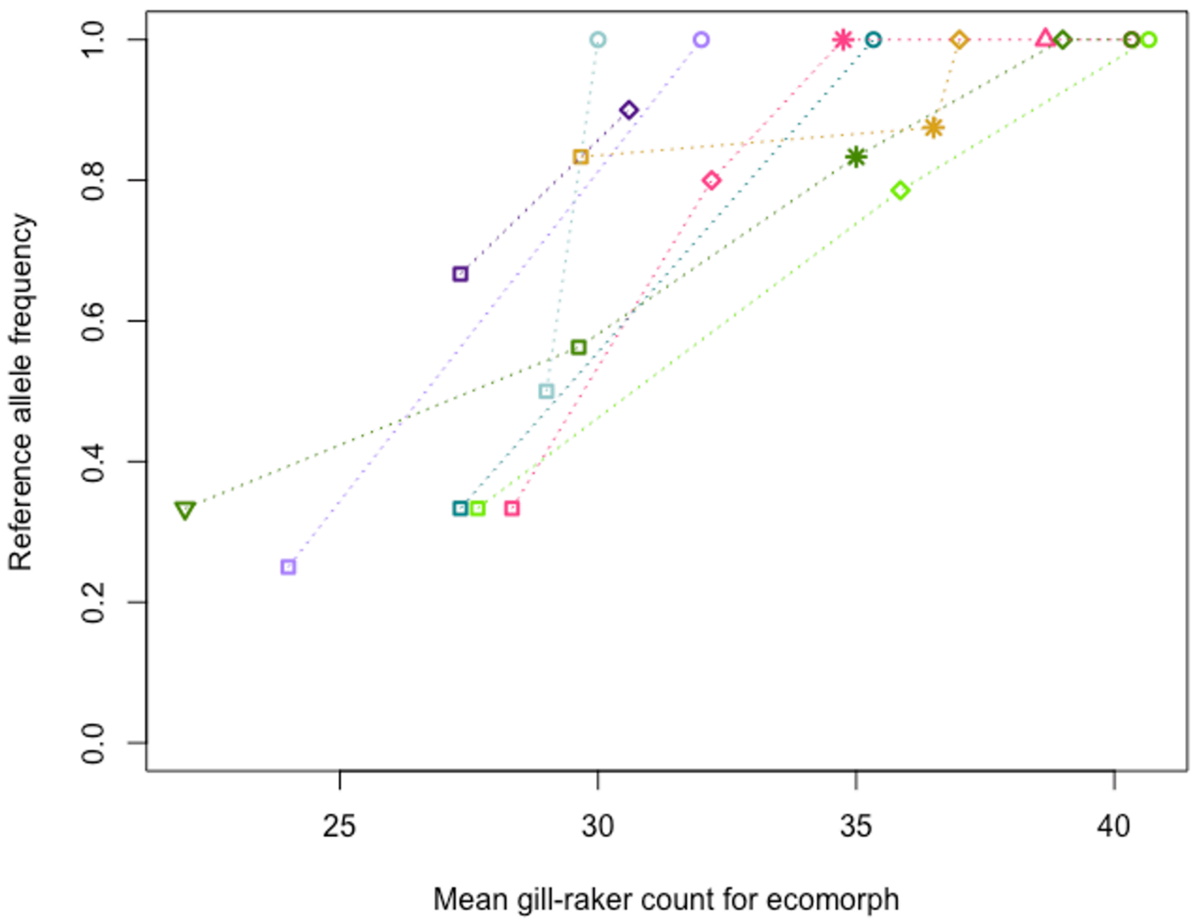

Supplementary Figure 9. Gill-raker count across species within each lake correlates to reference allele frequency for the significantly associated peak on WFS23. The reference allele frequency for each species split by lake and plotted against mean gill-raker count for those species individuals by lake. Only species with >1 individual were plotted (resulting in the exclusion of the one *C. heglingus* individual from lake Zurich). Symbols indicate ecomorph assignment and are used as in Supplementary Figure 1 and Figure 1.

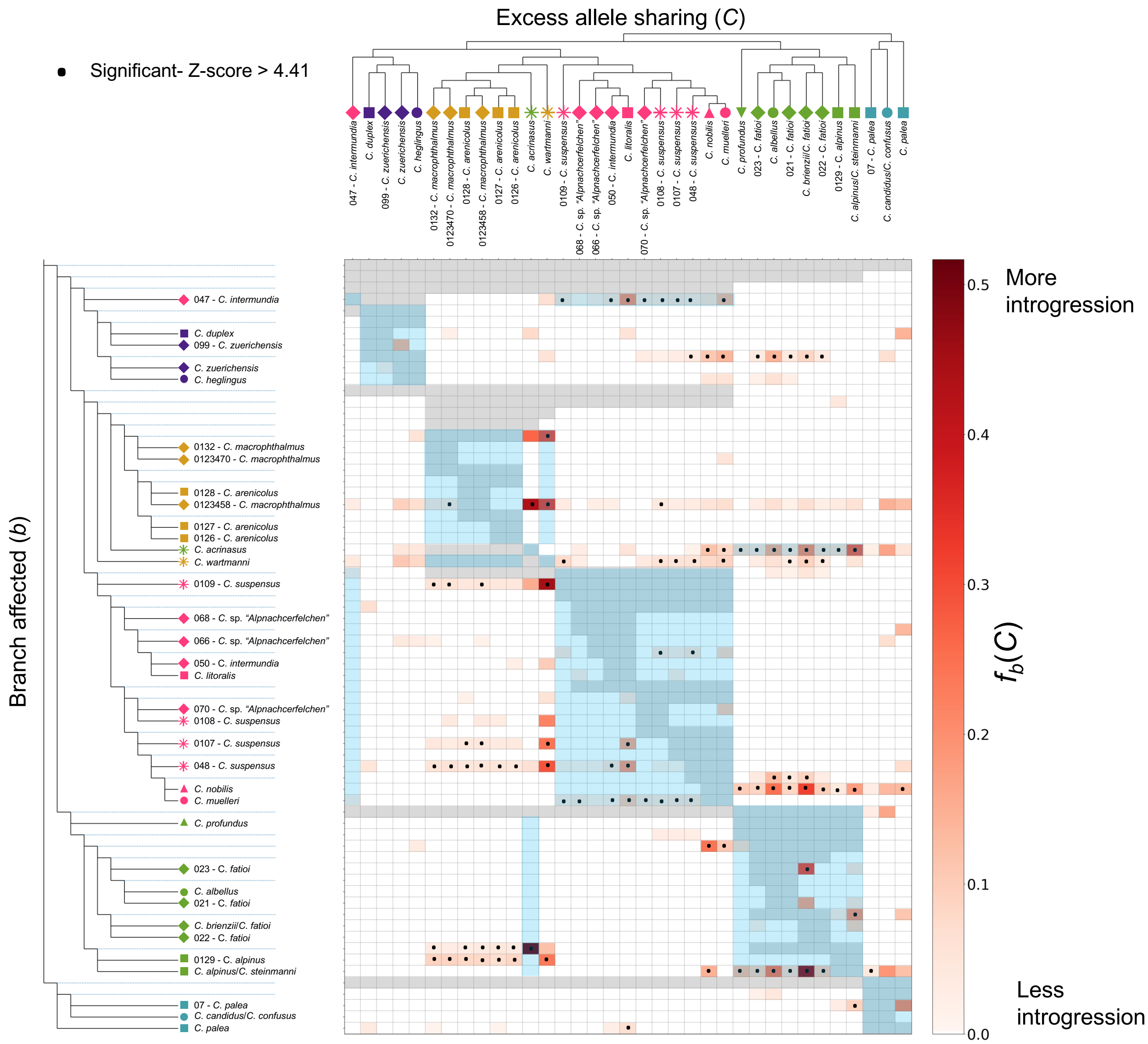

Supplementary Figure 10. Excess allele sharing is widespread between whitefish species both within and between lake-systems. F-branch ($f_b(C)$) statistics across our dataset highlight excess allele sharing between tips in the tree (which represent species or individuals when species were not monophyletic; horizontally arranged at the top of the figure) and each other tip (solid line) and node (dotted line) in the phylogenetic tree (vertically arranged on the left of the figure), compared to its sister branch. The associated lake and ecomorph of each tree tip is indicated by the symbol and colour (as in Supplementary Figure 1 and Figure 1a). The redness of each cell in the matrix indicates the degree of excess allele sharing between each tree tip (*C*) and each tip or node (*b*) with significant instances of excess allele sharing, where the Z-score was >4.41 (equivalent to the Bonferroni multiple testing corrected p-value of 0.01), are highlighted with a dot. For clarity, when a species within a lake or lake-system is supported as monophyletic we have collapsed all of its individuals into a single tree tip. Blue shading is used to indicate comparisons among species within a lake-system. Grey shading indicates tests which cannot be carried out due to the topology of the tree.

Supplementary Table 1. F4 values for each of the 12 topologies including Balchen and Albeli from lakes Brienz, Lucerne, Walen, Neuchâtel.

Topology for F4 test - (A,B),(C,D)	F4
(Balchen_Brienz,Albeli_Brienz),(Balchen_Neuchatel,Albeli_Neuchatel)	0.00325
(Balchen_Brienz,Albeli_Brienz),(Balchen_Lucerne,Albeli_Lucerne)	0.01021
(Balchen_Brienz,Albeli_Brienz),(Balchen_Walen,Albeli_Walen)	0.00868
(Balchen_Neuchatel,Albeli_Neuchatel),(Balchen_Lucerne,Albeli_Lucerne)	0.00732
(Balchen_Neuchatel,Albeli_Neuchatel),(Balchen_Walen,Albeli_Walen)	0.0056
(Balchen_Lucerne,Albeli_Lucerne),(Balchen_Walen,Albeli_Walen)	0.01272
(Balchen_Brienz,Balchen_Neuchatel),(Albeli_Brienz,Albeli_Neuchatel)	0.02063
(Balchen_Brienz,Balchen_Lucerne),(Albeli_Brienz,Albeli_Lucerne)	0.0238
(Balchen_Brienz,Balchen_Walen),(Albeli_Brienz,Albeli_Walen)	0.0248
(Balchen_Neuchatel,Balchen_Lucerne),(Albeli_Neuchatel,Albeli_Lucerne)	0.02067
(Balchen_Neuchatel,Balchen_Walen),(Albeli_Neuchatel,Albeli_Walen)	0.01697
(Balchen_Lucerne,Balchen_Walen),(Albeli_Lucerne,Albeli_Walen)	0.02035

Supplementary Table 2. Full results of statistical analysis of regressions between PC1 (calculated from loci within CSS windows) and gill raker count and standard length. Regressions of phenotypes across dataset.

**CSS PC1 vs. Gill-raker count**				
Lake system	DF	R^2	P	significance p < 0.001 = *** / p < 0.01 = ** / p < 0.05 = * / p > 0.05 = NS
All	88	0.3921	4.10E-11	***
All no outlier *C. profundus*	85	0.5107	7.63E-15	***
Luzern	16	0.8113	3.47E-07	***
Brienz/Thun including outlier *C. profundus*	31	0.3871	1.11E-04	***
Brienz/Thun no outlier *C. profundus*	28	0.6051	4.22E-07	***
Zurich/Walen	16	0.7167	9.43E-06	***
Biel/Neuchâtel	9	0.6857	0.001645	**
Constance	8	0.3703	0.06194	NS

**CSS PC1 vs. Standard length**				
Lake system	DF	R^2	P	
All	88	0.498	8.06E-15	***
Luzern	16	0.322	0.01405	*
Brienz/Thun	30	0.3547	0.0003233	***
Zurich/Walen	16	0.6925	1.84E-05	***
Biel/Neuchâtel	10	0.6738	0.001067	**
Constance	8	0.1215	0.3236	NS

**CSS PC1 vs. Gill-raker count (excluding original 24 samples)**				
All	64	0.1135	0.005667	**
All no outlier *C. profundus*	61	0.2201	0.000105	***

**CSS PC1 vs. Standard Length (excluding original 24 samples)**				
All	64	0.2081	0.0001183	***

GRC vs. Standard length (2 individuals with missing values excluded)	87	0.2767	1.20E-07	***

Supplementary Table 3. Details of genes, unique KEGG orthology terms, and KEGG pathways associated with outlier windows from across all four independent lake F_{ST} comparisons between 'Balchen' and 'Albeli' species (i.e. found at least once) and the numbers of each found in all four comparisons.

	Total present at least once across all four lakes	Number shared across all four lakes
Genes overlapping with top 1% F_{ST} outlier windows	1130	0
Unique KEGG orthology terms from these genes	660	2
KEGG pathways from these KEGG orthology terms	315	111
