# Supplementary material for: Genomic architecture of adaptive radiation and hybridization in Alpine whitefish
Source: Nat Commun. 2022 Aug 2;13:4479. doi: 10.1038/s41467-022-32181-8 (PMC9345977; doi:10.1038/s41467-022-32181-8)
Supplement: Supplementary file 2 — Description of Additional Supplementary Files [file 41467_2022_32181_MOESM2_ESM.pdf]

Description of Additional Supplementary Files

File Name: Supplementary Data 1
Description: All 99 whitefish sampled in this study including species name, lake of origin, ecomorph designation as well as phenotypic characteristics including standard length, gill-raker count, sex, and mean sequencing depth, as well as the unique lab individual ID and SRA accession number

File Name: Supplementary Data 2
Description: Significantly enriched gene ontology (GO) terms associated with genes overlapping the 342 outlier CS' windows according to both the 'weight' and 'elim' algorithms in topGO. GO terms are separated by category: cellular component (CC), biological process (BP) and molecular function (MF)

File Name: Supplementary Data 3
Description: Blastp results from parallel KEGG orthology terms: K07526 and K12959

File Name: Supplementary Data 4
Description: F-branch statistics across all Alpine whitefish species sampled.
