# Supplementary material for: Genomic architecture of adaptive radiation and hybridization in Alpine whitefish
Source: Nat Commun. 2022 Aug 2;13:4479. doi: 10.1038/s41467-022-32181-8 (PMC9345977; doi:10.1038/s41467-022-32181-8)
Supplement: Supplementary file 7 — Reporting Summary [file 41467_2022_32181_MOESM7_ESM.pdf]

nature portfolio

Corresponding author(s): PGDF

Last updated by author(s): Jul 11, 2022

Reporting Summary

Nature Portfolio wishes to improve the reproducibility of the work that we publish. This form provides structure for consistency and transparency in reporting. For further information on Nature Portfolio policies, see our Editorial Policies and the Editorial Policy Checklist.

Statistics

For all statistical analyses, confirm that the following items are present in the figure legend, table legend, main text, or Methods section.

n/a	Confirmed	
☐	☒	The exact sample size (*n*) for each experimental group/condition, given as a discrete number and unit of measurement
☐	☒	A statement on whether measurements were taken from distinct samples or whether the same sample was measured repeatedly
☐	☒	The statistical test(s) used AND whether they are one- or two-sided<br>*Only common tests should be described solely by name; describe more complex techniques in the Methods section.*
☒	☐	A description of all covariates tested
☐	☒	A description of any assumptions or corrections, such as tests of normality and adjustment for multiple comparisons
☐	☒	A full description of the statistical parameters including central tendency (e.g. means) or other basic estimates (e.g. regression coefficient) AND variation (e.g. standard deviation) or associated estimates of uncertainty (e.g. confidence intervals)
☐	☒	For null hypothesis testing, the test statistic (e.g. *F*, *t*, *r*) with confidence intervals, effect sizes, degrees of freedom and *P* value noted<br>*Give P values as exact values whenever suitable.*
☒	☐	For Bayesian analysis, information on the choice of priors and Markov chain Monte Carlo settings
☒	☐	For hierarchical and complex designs, identification of the appropriate level for tests and full reporting of outcomes
☒	☐	Estimates of effect sizes (e.g. Cohen's *d*, Pearson's *r*), indicating how they were calculated

Our web collection on statistics for biologists contains articles on many of the points above.

Software and code

Policy information about availability of computer code

Data collection	Illumina NovaSeq 6000 with a 550bp insert size (Next Generation Sequencing Platform, University of Bern)
Data analysis	The code used is available at https://github.com/RishiDeKayne/Alpine_whitefish_WGS<br>The code includes all open source software packages utilized as mentioned in the method section and listed here:<br>bwa-mem v.0.7.17<br>Mosdepth v.0.2.8<br>Picard-tools (Version 2.20.2; http://broadinstitute.github.io/picard/)<br>HaplotypeCaller in GATK v.4.0.8.1<br>vcftools v.0.1.14<br>BEDTools v.2.28.0<br>PLINK v.1.90<br>SAMtools v.1.8<br>vcf2phylip v.2<br>RAxML v.8.2.12<br>Figtree v.1.4.4<br>admixture v.1.3.0<br>ldPruning.sh (https://github.com/joanam/scripts/raw/master/ldPruning.sh)<br>plink2treemix.py (from https://speciationgenomics.github.io/Treemix/)<br>f4.py (https://raw.githubusercontent.com/mmatschiner/F4/master/f4.py)<br>CSS (https://github.com/marqueda/PopGenCode/blob/master/CSSm.R)<br>CSS permutation (https://github.com/marqueda/PopGenCode/blob/master/CSSm_permutation.R)<br>topGO package v2.46.0

BlastKOALA (https://www.kegg.jp/blastkoala/; using the taxon id 861768 and selecting the genus_eukaryotes database)
KEGG orthology database (https://www.kegg.jp/kegg/ko.html)
EMMAX v20120210
Ensembl TBLASTN (Ensembl release 106 - Apr 2022)
Dsuite v0.3

For manuscripts utilizing custom algorithms or software that are central to the research but not yet described in published literature, software must be made available to editors and reviewers. We strongly encourage code deposition in a community repository (e.g. GitHub). See the Nature Portfolio guidelines for submitting code & software for further information.

Data

Policy information about availability of data

All manuscripts must include a data availability statement. This statement should provide the following information, where applicable:

- Accession codes, unique identifiers, or web links for publicly available datasets
- A description of any restrictions on data availability
- For clinical datasets or third party data, please ensure that the statement adheres to our policy

The raw sequencing files are accessible on SRA (project ID PRJEB47792; accession numbers indicated in Table S1) and scripts are available on github (https://github.com/RishiDeKayne/Alpine_whitefish_WGS) . Additional supporting data (genotype file and corresponding meta data file) is deposited on the eawag research data institutional collections (https://doi.org/10.25678/0005S0).
The study further made use of the available whitefish reference genome (ENA accession: GCA_902810595.1).

Field-specific reporting

Please select the one below that is the best fit for your research. If you are not sure, read the appropriate sections before making your selection.

☐ Life sciences ☐ Behavioural & social sciences ☒ Ecological, evolutionary & environmental sciences

For a reference copy of the document with all sections, see nature.com/documents/nr-reporting-summary-flat.pdf

Ecological, evolutionary & environmental sciences study design

All studies must disclose on these points even when the disclosure is negative.

Study description	Whole genome sequencing of 99 whitefish spanning six distinct ecomorphs across five independent pre-Alpine lake-systems.
Research sample	The sex and phenotypic information for the individual samples is given in Supplementary Data 1. Organisms were not manipulated.
Sampling strategy	We focused our study on representing the diversity across a species rich radiation and decided to sample three individuals for each species present in one of the sampled lakes. We sampled Corgonus spp across pre-Alpine lakes. Lakes were chosen to represent pre-Alpine lakes documented to harbour at least two sympatric species. In each lake sampled all described species for the lake were sampled.
Data collection	Illumina NovaSeq 6000 with a 550bp insert size (Next Generation Sequencing Platform, University of Bern)
Timing and spatial scale	The samples for individual genomic sequencing were collected between 2014 and 2018 and across five independent pre-Alpine lake-systems (lakes Constance, Thun/Brienz, Lucern, Biel/Neuchatel, Walen/Zurich).
Data exclusions	No data was excluded from the analysis.
Reproducibility	Genomic sequencing of individual samples was not replicated.
Randomization	Randomization is not applicable to genomic sequencing of individuals.
Blinding	Blinding is not applicable to genomic sequencing of indivdiuals.

Did the study involve field work? ☐ Yes ☒ No

Reporting for specific materials, systems and methods

We require information from authors about some types of materials, experimental systems and methods used in many studies. Here, indicate whether each material, system or method listed is relevant to your study. If you are not sure if a list item applies to your research, read the appropriate section before selecting a response.

Materials & experimental systems

n/a	Involved in the study
☒	☐ Antibodies
☒	☐ Eukaryotic cell lines
☒	☐ Palaeontology and archaeology
☐	☒ Animals and other organisms
☒	☐ Human research participants
☒	☐ Clinical data
☒	☐ Dual use research of concern

Methods

n/a	Involved in the study
☒	☐ ChIP-seq
☒	☐ Flow cytometry
☒	☐ MRI-based neuroimaging

Animals and other organisms

Policy information about studies involving animals; ARRIVE guidelines recommended for reporting animal research

Laboratory animals	No laboratory animals were used.
Wild animals	Individuals used were caught by local fishermen during the spawning season and during a comprehensive field sampling campaign. Individuals were anaesthetized and subsequently euthanized using appropriate concentrations of tricaine methane sulfonate solutions (MS-222) according to the permit issued by the cantons of Zurich (ZH128/15), Bern (BE68/15), and Lucerne (LU04/14).
Field-collected samples	No laboratory animals were used.
Ethics oversight	Permit issued by the cantons of Zurich (ZH128/15), Bern (BE68/15), and Lucerne (LU04/14).

Note that full information on the approval of the study protocol must also be provided in the manuscript.
